# Preparation of 2D Carbon Materials by Chemical Carbonization of Cellulosic Materials to Avoid Thermal Decomposition

**DOI:** 10.1002/gch2.201700061

**Published:** 2017-08-22

**Authors:** Mutsumasa Kyotani, Kazuhisa Hiratani, Tatsuhiro Okada, Satoshi Matsushita, Kazuo Akagi

**Affiliations:** ^1^ Conjugated Polymer Super‐Hierarchical Control Lab Katsura Int'tech Center 2F‐212 Kyoto University Katsura Kyoto 615‐8510 Japan; ^2^ Department of Applied Chemistry Utsunomiya University Utsunomiya 321‐8582 Japan; ^3^ Tsukuba Fuel Cell Laboratory, Inc. Shimotakatsu 2‐14‐3 Tsuchiura Ibaraki 300‐0812 Japan; ^4^ Department of Polymer Chemistry Kyoto University Katsura Kyoto 615‐8510 Japan

**Keywords:** carbonization reactions, fabrics, high carbon yields, papers, tough carbon materials

## Abstract

Cellulosic materials, including regenerated cellulose, are promising precursors for a variety of carbon materials. However, thermal decomposition, typically accompanying carbonization at high temperatures, hinders cellulosic materials from being efficiently carbonized (i.e., very low carbon yields). Herein, this study presents a new and efficient method for the preparation of porous 2D carbon materials from sheet‐like cellulosic materials, such as papers and fabrics, involving a catalyzed chemical reaction at high temperatures without thermal decomposition. Thus, cellulosic materials are treated with sulfonic acid solutions and significantly dehydrated at high temperatures via evaporation of water. As a result, black materials are obtained at a weight near the theoretical carbon content of cellulose and remain in the carbonized materials. The as‐obtained porous 2D carbon materials are flexible and suitable for a wide range of applications such as in electrodes and gas absorbents.

Cellulosic materials have many advantages including being abundant in nature, having a renewable character, and being easy to process. These materials are used in a variety of forms such as fiber, cloth, and paper.^1^, ^2^ Regenerated cellulosic materials chemically derived from natural celluloses are also useful.^2^ On the other hand, cellulose‐based carbon materials composed of carbon with hexagonal‐bond sheets like graphene can be potentially used in numerous fields such as electrochemical energy storage^3^, ^4^ and medicine of the medical field.^5^ The study of the efficient synthesis (i.e., high carbon yield) of carbon materials from cellulosic sources is attractive and challenging.

Cellulosic materials are usually carbonized via pyrolysis at high temperatures.^6^, ^7^ Cellulose molecules are known to thermally decompose during the pyrolysis process at high temperatures, thereby giving rise to complex degradation reactions to form low molecular weight compounds such as levoglucosan and subsequent formation of volatile hydrocarbon gases with lower molecular weights.^8^ As a result, the final carbon yield (referred to as the source cellulose material) is usually less than 20 wt%.^9^ Furthermore, carbonized cellulosic materials are very fragile and highly prone to form small black particles. Thus, low carbon yield and weakness are characteristics of carbonized cellulosic materials obtained by pyrolysis.

With the aim to enhance the carbon yield, carbonization of cellulosic materials has been performed in the presence of numerous inorganic acids (e.g., phosphoric acid) and transition metals via impregnation.^10^ A stabilization process by which cellulosic materials are oxidized in air at a temperature lower than 300 °C is often performed before carbonization.^11^ However, regardless of the impregnation and stabilization processes, thermal decomposition typically accompanies carbonization of cellulosic materials at high temperatures, thereby limiting the carbon yield.^12^

Iodine has been recently used as an effective catalyst for carbonization of some precursors such as coal tar pitch^13^, ^14^ and polyacetylene films.^15^, ^16^ We showed that iodine‐treated helical polyacetylene films can almost be fully carbonized without thermal decomposition while retaining their initial morphology.^15^, ^16^ Furthermore, we have reported that cellulosic papers treated with iodine can be carbonized with a little thermal decomposition with carbon yields up to 30 wt%.^17^

Herein, we present a novel chemical carbonization method without thermal decomposition at high temperatures applicable to most of cellulosic materials. The morphological properties of the cellulosic materials (i.e., nanometric size of the fibers) remained nearly constant after the carbonization. In this report, three kinds of sheet‐like cellulosic materials, i.e., a paper made from sisal fibers and two fabrics made from cotton threads and rayon filaments (Table S1, Supporting Information), were used as precursors for preparing porous 2D carbon materials. The fibrous structures of the paper, as well as textural properties of the fabrics, were retained after the carbonization, although total shrinks in both sheet‐size and thickness occurred to some extent. Remarkably, the carbon yield was higher than 34 wt%, which was close to the carbon content of cellulose (44.4 wt%).

Numerous organic sulfonic acids can be used as catalysts in the carbonization reaction. In particular, methane sulfonic acid (MSA) is the most effective catalyst. In this work, sheet‐like cellulosic materials were treated with an MSA solution before carbonization, as indicated in the Experimental Section.

The thermal behavior, while heating three sheet‐like cellulosic materials before and after the MSA treatment, is shown in **Figure** **1** as thermogravimetric (TG), derivative thermogravimetric (DTG), and differential thermal analysis (DTA) curves.

**Figure 1:**
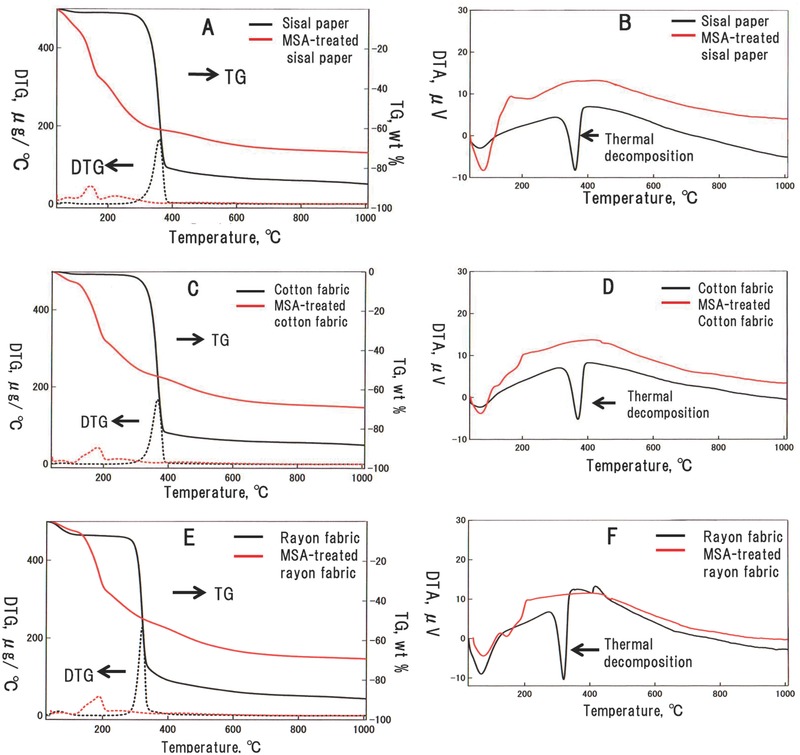
Thermal behavior of the sheet‐like cellulosic materials while heating up to 1000 °C at a heating rate of 10 °C min^−1^ under flowing nitrogen gas. A,C,E) TG/DTG and B,D,F) DTA curves for A,B) the sisal paper, C,D) the cotton fabric, and E,F) the rayon fabric. The black and red curves in the figure correspond to the source cellulosic materials and the MSA‐treated cellulosic materials, respectively. The thermal decomposition indicated by an arrow in each DTA curve corresponds to the endothermic decomposition peak.

Before the treatment, all the specimens showed TG curves with a large weight loss from 300 to 400 °C accompanied with a large endothermic peak (indicated by an arrow) clearly detected at 350–370 °C in the DTA curves (Figure 1). These features (i.e., large weight loss and large endothermic peak) were mainly attributed to the thermal decomposition and gasification of the cellulosic materials.^18^, ^19^, ^20^, ^21^, ^22^, ^23^, ^24^

On the other hand, as shown in the TG and DTG curves in Figure 1, the MSA‐treated cellulosic materials showed lower weight losses than the pristine materials starting at room temperature. Moreover, no large endothermic peaks were observed in the DTA curves for the MSA‐treated cellulosic materials (Figure 1), thereby suggesting that the MSA absorbed plays a role in preventing the cellulosic materials from thermal decomposition while simultaneously avoiding the evaporation of organic gases even at high temperatures, as described below. As a result, the carbon yield increased because the carbon atoms contained in the cellulose molecules remained in the carbonized materials.

Numerous gases are released during carbonization of the cellulosic materials,^8^, ^24^, ^25^ especially, in the temperature range of the large weight loss observed in the TG curves described above. The main gases released during the heating process are listed in Table S2 in the Supporting Information for all the cellulosic materials used in this work. The amount of gases released up to 500 °C for the cellulosic materials before and after the MSA treatment is shown in **Figure** **2**. Evaporated water was the main gas released from the samples.^24^, ^26^, ^27^ However, when the specimens before and after the MSA treatment were compared, the amount of evaporated water remarkably increased for all the MSA‐treated cellulosic materials, as shown in Figure 2. Moreover, the amount of released organic gases containing elemental carbon such as acetic acid notably decreased for the MSA‐treated cellulosic materials, as shown in Figure 2 and Table S2 in the Supporting Information.

**Figure 2:**
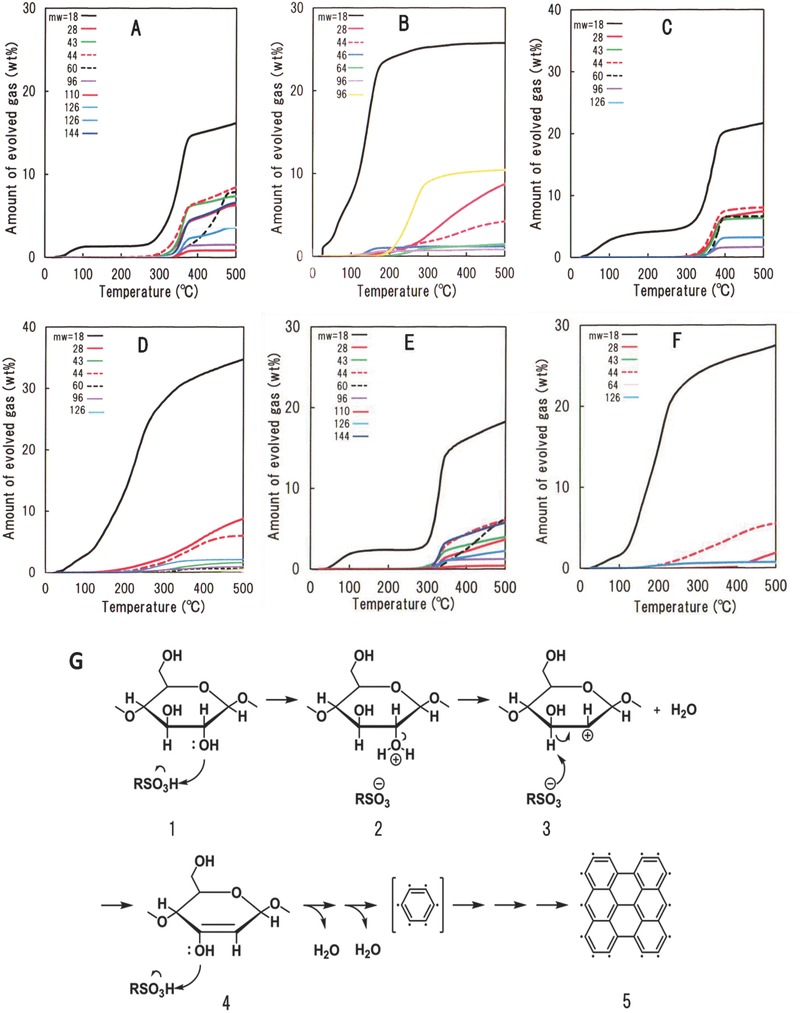
Integrated curves (A–F) of the released gases as a function of temperature in a heating run up to 500 °C under flowing argon gas for the sheet‐like cellulosic materials, and dehydration reaction scheme from a glucose residue unit (G). Curves (A and B) correspond to the sisal paper and its MSA‐treated counterpart, curves (C and D) refer to the cotton fabric and its MSA‐treated counterpart, and curves (E and F) refer to the rayon fabric and its MSA‐treated counterpart. The number, *m*_w_, shown in (A–F) stands for the molecular weights of released gases described in Table S2 in Supporting Information.

The above results of gases released while heating the samples (TG curves in Figure 1) indicated that the observed weight losses were, to some extent, produced by the evaporation of water from the cellulosic materials before the MSA treatment, whereas a fraction of this weight loss was produced by the release of some organic gases. All MSA‐treated cellulosic materials continuously released large amounts of evaporated water along with very low amounts of carbon atoms while heating from room temperature. Therefore, these results suggested that the large weight loss observed for all of MSA‐treated cellulosic materials (Figure 1) is mainly produced by the evaporation of large amounts of water from the cellulosic materials. MSA likely catalyzed the dehydration of cellulosic molecules in an effective manner.

The analysis of the gases released from the MSA‐treated cellulosic materials during the carbonization process confirmed that the major weight loss observed in the TG curves was mainly produced by the evaporation of water accompanying dehydration of cellulose molecules. The carbonization process of the sulfonic acid‐treated cellulosic materials at high temperatures is complex and difficult to clear in detail at this stage, though thermal decomposition almost does not occur. However, on the basis of the observations of the notable dehydration reaction and the remarkable decrease of organic gases containing carbon atoms releasing during the carbonization process, as described above, the carbonization process is believed to be the scheme illustrated in Figure 2G. As the carbonization scheme of the sulfonic acid treated cellulosic materials is fully additive, the dehydration reaction starts at first. The sulfonic acid (RSO_3_H, MSA in this work) reacts with the hydroxyl and ether linkages in the glucose residue of cellulose (Figure 2G1,2) to perform dehydration. It is supposed that the proton of the considerably strong organic sulfonic acid catalyst randomly attacks both hydroxyl and ether oxygen atoms thereby breaking the C=O bonds followed by dehydration (Figure 2G3,4) of the polymeric glucose chains to form carbon materials. The dehydration finally results in the formation of a carbon hexagonal bond unit that subsequently combines in 2D, similar to graphene (Figure 2G5) having numerous free radicals, as revealed by electron‐spin resonance (ESR) measurements (Section S6, Supporting Information).

The high efficiency of organic sulfonic acids in avoiding carbon losses as compared to the numerous dehydration catalysts used so far is supposed to lie in the appropriate acidity and homogenous dispersion of sulfonic acid on the cellulosic materials as a result of its characteristic of fluidity. Thus, smooth dehydration and recombination of carbon—carbon bonds are promoted, resulting in the continuous formation of carbon hexagonal bond units, as shown in Figure 2G.

As mentioned above, iodine is one of the effective catalysts. However, sulfonic acids can disperse more homogenously on the cellulosic materials as compared with iodine even in the crystalline regions. As a result, the dehydration reaction progresses uniformly in the samples at high temperatures. Moreover, both iodine and sulfonic acids are well‐known compounds that exhibit a leaving group activity in the organic chemistry field. Thus, it is inferred that the compounds that have leaving groups can act as effective catalysts to avoid thermal decomposition in the carbonization of polymeric materials.

The effects of MSA treatment on the kinetics of the carbonization reaction for cellulosic materials were examined using the TG curves (Figure 1), as described in Section S4 in the Supporting Information. The apparent activation energy for the carbonization reaction, calculated from the analysis of TG curves (Figure 1), remarkably decreased for the samples undergoing the MSA treatment, as shown in Table S3 in the Supporting Information. This result suggested that the MSA contained in the cellulosic materials worked as a catalyst, markedly decreasing the energy barrier for the carbonization reaction in these materials.

Pictures and scanning electron microscopy (SEM) images of surfaces structures are shown in **Figure** **3** for the MSA‐treated sheet‐like cellulosic materials before and after carbonization. The fibrous structures of the carbonized paper were similar to those of the source material (sisal paper). The textural structures of both cotton and rayon fabrics (woven with threads or filaments) were nearly preserved after the carbonization process, although an individual fibril, a filament, and thread shrank in thickness to some extent. All the sheet‐like cellulosic materials treated with MSA shrank by ≈50% in total size and by ≈70% in thickness upon carbonization at 800 °C. The MSA‐treated sheet‐like cellulosic materials were transformed into porous 2D carbon materials, a carbon paper and two kinds of carbon fabrics, after carbonization (Figure 3) since the source sheet‐like cellulosic materials were porous. However, the pore size distribution changed to some extent after carbonization. The pore size distribution curves of the 2D carbon materials are shown in Figure S5 in the Supporting Information.

**Figure 3:**
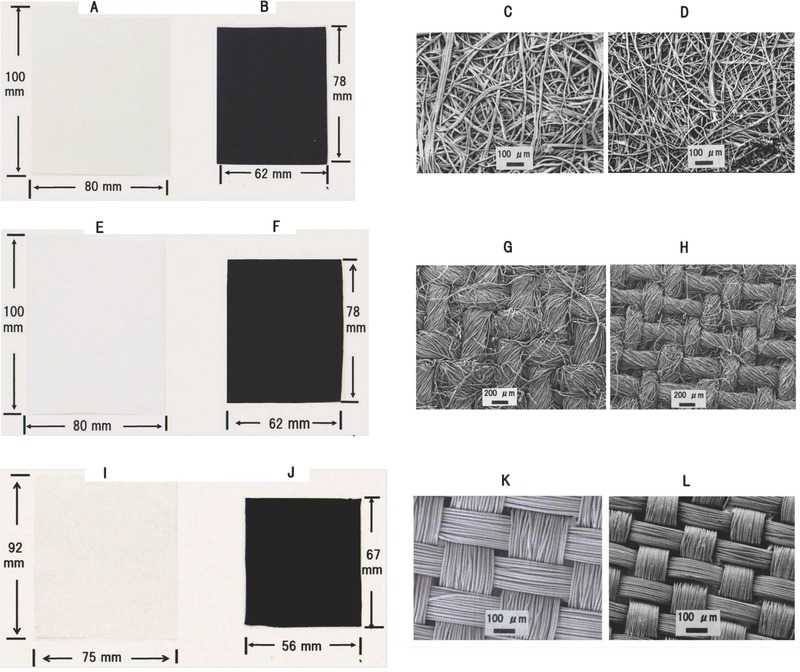
A,B,E,F,I,J) Pictures and C, D, G, H, K, and L) SEM images of the sheet‐like cellulosic materials before and after carbonization. The source materials (A and C: sisal paper, E and G: cotton fabric, and I and K: rayon fabric) were carbonized at 800 °C in an argon atmosphere after the MSA treatment. The carbonized materials (B and D, F and H, and J and L, corresponding to A and C, E and G, and I and K, respectively) shrank in macroscopic (B, F, and J) and in microscopic (D, H, and L) sizes. The scales of the figure show the actual sizes.

The X‐ray diffraction (XRD) patterns of the 2D carbon materials before and after the heat‐treatment described in the Experimental Section are shown in **Figure** **4**. All the 2D carbon materials prepared at 800 °C showed no crystalline reflections (Figure 4). It is believed that the 2D carbon materials prepared at 800 °C have the so‐called turbostratic structure.^28^ However, a diffraction peak at a distance of 0.34 nm corresponding to the (002) face of a graphitic crystal^28^ was observed for carbon materials heat‐treated above 2200 °C, and the intensity of this peak increased with the temperature of the heat‐treatment (Figure 4). The elemental analysis showed that all the 2D carbon materials heat‐treated at temperatures above 1800 °C were formed by pure carbon. This result indicated that the carbonization of the sheet‐like cellulosic materials was completed at temperatures above 1800 °C.

**Figure 4:**
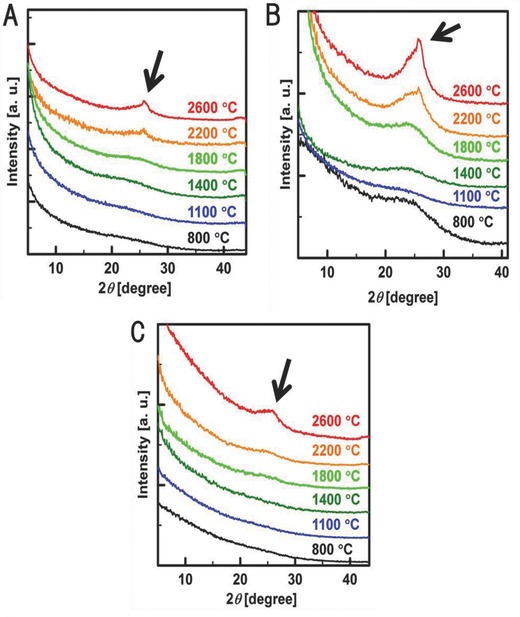
XRD patterns of the 2D carbon materials prepared from the MSA‐treated sheet‐like cellulosic materials (A: sisal paper, B: cotton fabric, C: rayon fabric). Carbonization was performed at 800 °C in an argon atmosphere after the MSA treatment. The materials carbonized at 800 °C (the bottom curves in A, B, and C) were heat‐treated at the temperatures indicated in the figure for 30 min in an argon atmosphere. The small diffraction peaks indicated by arrows in the figure correspond to the (002) face of graphitic crystals.

The Raman scattering spectra, mechanical, and electrical properties of the 2D carbon materials are described in the Sections S7–9 in the Supporting Information, respectively. Remarkably, the carbon materials prepared from the MSA‐treated rayon samples showed notably high elastic modulus and strength values. An example of the application of the porous 2D carbon materials as a fuel cell electrode is shown in the Section S11 in the Supporting Information.

In summary, this paper reported that handy porous 2D carbon materials were prepared from MSA‐treated sheet‐like cellulosic materials via a chemical carbonization method without thermal decomposition at high temperatures. Carbon yields close to the theoretical carbon content of cellulose were obtained. The graphitic structures, mechanical properties, and electrical conductivity of the 2D carbon materials were enhanced by the heat‐treatment at higher temperatures.

## Experimental Section

Three types of sheet‐like cellulosic materials, namely, a paper made from sisal fibers and two fabrics made from cotton yarns and cupra rayon filaments, were used in this work.

Sisal papers were made of fibers extracted from raw leaves, as described in the Section S1 in the Supporting Information. The two fabrics were commercial samples. The cotton fabric had a twill weave construction (44 and 22 yarns per 10 mm of warp and weft, respectively, and 20 in tex value per yarn) and was obtained from Kiyohara Co. (Osaka, Japan). The rayon fabric had a plain weave construction (50 and 36 filaments per 10 mm of warp and weft, respectively, comprising 20 filaments with each one having a tex value of 20) was obtained from Asahi KASEI Co.

The thickness, basal weight, water content, and elemental analysis data of the cellulosic materials used in this work are listed in Table S1 in the Supporting Information.

The treatment of sheet‐like cellulosic materials with MSA for the preparation of 2D carbon materials was as follows. The cellulosic materials were immersed in a 1 m MSA aqueous solution at room temperature for 5 min to allow the absorption of MSA by the cellulosic materials. After the treatment, the cellulosic materials were separated from the solution and subsequently dried at room temperature to remove excess water until it attained near constant weight. The relative humidity in the laboratory was 40–70%. The amount of absorbed MSA was determined by comparing the weight of the cellulosic materials before and after the treatment. In this study, the amounts of absorbed MSA were 50–70 and 10–30 wt% for the sisal paper and both cotton and rayon fabrics, respectively.

The carbonization behavior of the cellulosic materials before and after the MSA treatment was investigated by TG/DTA of the samples (8–10 mg) up to 1000 °C at a rate of 10 °C min^−1^ under flowing nitrogen gas on a TG/DTA6200 (Seiko Instrument) apparatus. The DTG curves were obtained by differentiating the corresponding TG curves. This study attempted to determine the apparent activation energy of carbonization using the TG data with the aim of examining the effect of the MSA treatment on the carbonization kinetics, as mentioned in the Section S4 in Supporting Information.

Furthermore, to investigate the reaction mechanism for the carbonization of the cellulosic materials before and after the treatment in detail, the gases released while heating the samples were analyzed by mass spectrometric (MS) analysis on a GC/MS QP2010 apparatus from Shimadzu. The sensitivity of the MS apparatus was 1.80 kV and the sample weight used for the GC/MS analysis was ≈1 mg. The mass‐to‐charge ratio (*m*/*z*) ranged from 10 to 300. Here *m* and *z* stand for the mass number and the ionic valence, respectively. Here, the ionic valence was considered to be one. The gases released while heating the samples at a rate of 6 °C min^−1^ up to 500 °C under flowing helium gas (50 mL min^−1^) were examined. The distribution curve of a released gas in wt% per second was obtained as a function of temperature using the mass‐number strength curve at the given temperature.

The sheet‐like cellulosic materials were held between two carbon plates, heated to 800 °C using an electric furnace (KDF75, Denken) at a rate of 6 °C min^−1^, and were subsequently kept at 800 °C for 1 h under flowing argon gas to carbonize them. After carbonization, the electric furnace was cooled to room temperature, and the carbonized sheet‐like materials (2D carbon materials) obtained were taken out of the furnace. It is necessary to pay attention to the flowing gas leaving the furnace during the carbonization of MSA‐treated samples at high temperatures because the gas contains a smelly MSA gas (Table S2, Supporting Information). Active carbon particles were used to capture the MSA gas.

The 2D carbon materials were heat‐treated at temperatures ranging from 1100 to 2600 °C for 30 min under flowing argon gas in a graphitizing apparatus (Sanriko Denki). The heating rate of the heat‐treatment temperature was ≈30 °C min^−1^.

The structural properties of the 2D carbon materials, including the heat‐treated samples, were investigated by XRD (RINT2500, Rigaku) with Ni‐filtered Cu Kα radiation (λ = 0.154 nm) and by SEM at an accelerating voltage of 5 kV (Topcon DS‐130).

The ESR spectra, the Raman scattering spectra, the mechanical and electrical properties, and the microporous structures of the 2D carbon materials are described in the Supporting Information.

## Conflict of Interest

The authors declare no conflict of interest.

## Supporting information

Supplementary
