# Supplementary material for: Preparation of 2D Carbon Materials by Chemical Carbonization of Cellulosic Materials to Avoid Thermal Decomposition
Source: Glob Chall. 2017 Aug 22;1(7):1700061. doi: 10.1002/gch2.201700061 (PMC6607140; doi:10.1002/gch2.201700061)
Supplement: Supplementary file 1 — Supplementary [file GCH2-1-1700061-s001.pdf]

Copyright WILEY-VCH Verlag GmbH & Co. KGaA, 69469 Weinheim, Germany, 2017.

Global Challenges
Open Access

Supporting Information

for *Global Challenges,* DOI: 10.1002/gch2.201700061

Preparation of 2D Carbon Materials by Chemical Carbonization of Cellulosic Materials to Avoid Thermal Decomposition

Mutsumasa Kyotani, Kazuhisa Hiratani, Tatsuhiro Okada, Satoshi Matsushita, and Kazuo Akagi*

Copyright WILEY-VCH Verlag GmbH & Co. KGaA, 69469 Weinheim, Germany, 2016.

Supporting Information

Preparation of Two-dimensional Carbon Materials by Chemical Carbonization of Cellulosic Materials to avoid Thermal Decomposition

Mutsumasa Kyotani, Kazuhisa Hiratani, Tatsuhiro Okada, Satoshi Matsushita, Kazuo Akagi*

S1. Preparation of the sisal paper

The sisal papers were made of fibers extracted from raw leaves produced in Ecuador. The extracted fibers were cut into short pieces of less than 3 mm in length with a cutting machine. The short sisal fibers were placed in a sodium hydroxide/water solution and boiled for 7 h to extract the lignin fraction. The fibers were subsequently refined using a beating machine for 2 h to prepare a paper pulp, bleached in a sodium hypochlorite solution for 30 min, and finally rinsed with water.[1, 2] The freeness of the obtained pulp was 735 CSF.[3] The sisal papers used in this work were prepared from the obtained pulp using a paper-making machine (Kumagai Riki Kogyo Co., Japan) equipped with an 80 mesh network.

S2. Characterization of the sheet-like cellulosic materials

The thickness, basic weight, and water content of the sheet-like cellulosic materials were measured using a thickness meter (Litematic VL-50A, Mitsutoyo), a balance meter (AB-135-S, Mettler Toledo), and a moisture meter (MX-50, A & D), respectively.

Elemental analysis was carried out on the cellulosic materials using a Yanaco CHN Corder MT-5 in the Microanalysis Center of the Kyoto University. The absolute weight percentages of elemental carbon, hydrogen, and nitrogen in a determined specimen were measured using this apparatus. Other elements were not analyzed herein.
The data are summarized in Table S1.

Table S1. Characterization of the sheet-like cellulosic materials.

	Thickness (μm)	Basic weight (g/m^2)	Water content (wt. %)	Elemental analysis (wt %)		
				Carbon	Hydrogen	Nitrogen
Sisal paper	378	144	7.0	41.7	6.1	0.0
Cotton fabric	312	196	8.5	42.2	6.2	0.0
Rayon fabric	83	65	11.0	40.0	6.2	0.4

1.Thickness was determined as the average value obtained from the measurement of 100 points of a 300 mm × 300 mm specimen.

2. Basic weight was determined as the average value calculated from the weights of 10 specimens 80 mm × 100 mm in size and the measured thickness.

S3. Analysis of the gasses released while heating during the carbonization of the sheet-like cellulosic materials before and after the MSA-treatment

The main gasses released while heating up to 500°C during the carbonization process are listed (wt% with regard to the source specimens) in Table S2. These gasses were identified on the basis of the MS curves obtained, as mentioned in the Experimental section of the main text.

The mark × in Table S2 indicates that the corresponding gas was not identified by the apparatus used herein, while n.d. indicates that the corresponding gas was not detected.

In the case of the sheet-like cellulosic materials treated with MSA, water vapor was released while heating from room temperature. The dehydration reaction is likely to begin at room temperature. Therefore, it is believed that the amount of water released is larger by several wt% units than that shown in Table S2 for all of the MSA-treated cellulosic materials. This difference was inferred from the difference in the weight of the samples set in a chamber, and those immediately after the MS measurements were initiated after attaining an appropriate He gas atmosphere in the chamber. The weight of the latter samples slightly decreased as compared with the former samples.

Release of carbon monoxide and carbon dioxide was observed from all the cellulosic materials, irrespective of the MSA treatment.

The release of an aldehyde gas species having a molecular weight (MW) of 43 was observed to a lower extent after the MSA treatment for all the materials.[4]

Formic acid gas was detected exclusively for the MSA-treated sisal paper samples.

The gasses having a MW of 60 showed two components, which were difficult to separate with the device used herein.

All the MSA-treated cellulosic materials generated two gasses: sulfur dioxide and MSA. In particular, large amounts of MSA were released by the MSA-treated sisal paper likely because of the larger amounts of absorbed MSA present in this sample versus the other cellulosic materials, as shown in Table S2.

Maltol gas was identified only for the sisal paper samples.

Table S2. Main gasses released while heating the samples up to 500°C during the carbonization of the sheet-like cellulosic materials before and after the MSA treatment.

Molecular weight	Assignment of gas	Sisal paper		Cotton fabric		Rayon fabric	
		Original	MSA-treated	Original	MSA-treated	Original	MSA-treated
		wt %	wt %	wt %	wt %	wt %	wt %
18	Water	16.2	25.7	22.0	35.0	18.0	27.0
28	Carbon monoxide	6.3	8.7	7.4	8.8	3.7	1.8
43	An aldehyde ($OCH\text{-}CH_2$-)	7.3	×	6.3	1.6	4.0	0.7
44	Carbon dioxide	8.5	4.2	8.0	6.0	6.1	5.5
46	Formic acid	×	1.2	×	×	×	×
60	Acetic acid and Glycoaldehyde	7.9	n.d.	6.6	0.6	6.3	0.1
64	Sulfur dioxide	n.d.	1.4	n.d.	0.5	n.d.	0.6
96	Furancarboxaldehyde	1.5	0.8	1.6	n.d.	1.2	n.d.
96	Methane-sulfonic acid	×	10.4	×	0.9	×	0.3
110	Methy-furancarboxaldehyde	0.8	×	×	×	0.4	×
126	Levoglucosenone	3.6	×	3.2	2.1	2.2	0.7
126	Maltol	3.6	×	×	×	×	×
144	Dianhydro- α -d-glucopyranose	6.6	×	×	×	5.7	0.6

S4. Carbonization kinetics of the sheet-like cellulosic materials

The effect of the absorbed MSA on the carbonization kinetics of the sheet-like cellulosic materials used herein was examined using the TG curve data shown in Figure 1. Although the carbonization kinetics of cellulose fundamentally involve a single reaction.[4, 5, 6] as described above, numerous gasses were released while heating during the carbonization process and can be expressed assuming a given heating rate by the following equations:

$$d\alpha/dT = (d\alpha/dt)\,(dt/dT) \qquad (1)$$

$$\alpha = (W_0 - W)/\,(W_0 - W_f\,) \qquad (2)$$

where α is the degree of conversion, t and T denote time and temperature, respectively, dt/dT represents the inverse of the heating rate, W is the mass of the substrate present at any time t, W_0 is the initial substrate mass, and W_f is the final mass of solids remaining after carbonization. In a first-order reaction, the reaction rate is proportional to the fraction of unreacted substrate as follows:

$$d\alpha/dT = k\,(1 - \alpha) \qquad (3)$$

where k is the rate constant ordinarily expressed by the following Arrhenius equation:

$$\ln k = \ln A - E_a/RT \qquad (4)$$

where A is the frequency factor, R is the universal gas constant, and E_a is the apparent activation energy.

The rate constant k can be calculated by analyzing the TG curves shown in Figure 1. Figure S1 shows the Arrhenius plot for the carbonization reaction of the pristine cellulosic materials (Figure S1 (I)) and those undergoing the MSA treatment (Figure S1 (II)). Table S3 summarizes the values of E_a obtained from the slopes of the straight lines versus the inverse

of the temperature shown in Figure S1. The apparent activation energy for cellulose carbonization has been widely reported.[4–9] The cellulosic materials before the MSA treatment showed E_a values (Table S3) in the range of those previously reported for the carbonization of cellulosic materials.[4–9]

However, it should be noted that two or three different values of E_a were obtained for the carbonization of MSA-treated sheet-like cellulosic materials, depending on the rate constant obtained from the analysis of the corresponding TG curves, as shown in Figure S1 and Table S3. The MSA-treated materials showed remarkably lower E_a values for the carbonization process as compared with the pristine samples. This result suggests that the absorbed MSA acts as a carbonization catalyst. The absorbed MSA likely promotes dehydration of cellulose molecules during heating and simultaneously suppresses the thermal decomposition of the cellulosic materials. The different values of E_a obtained for the MSA-treated cellulosic materials mentioned above are believed to reflect the release of different gasses depending on the temperature during the heating run.

The amount of released carbon containing organic gasses remarkably decreased for the MSA-treated cellulosic materials. As a result, the carbon yield increased during the carbonization of the MSA-treated cellulosic materials.

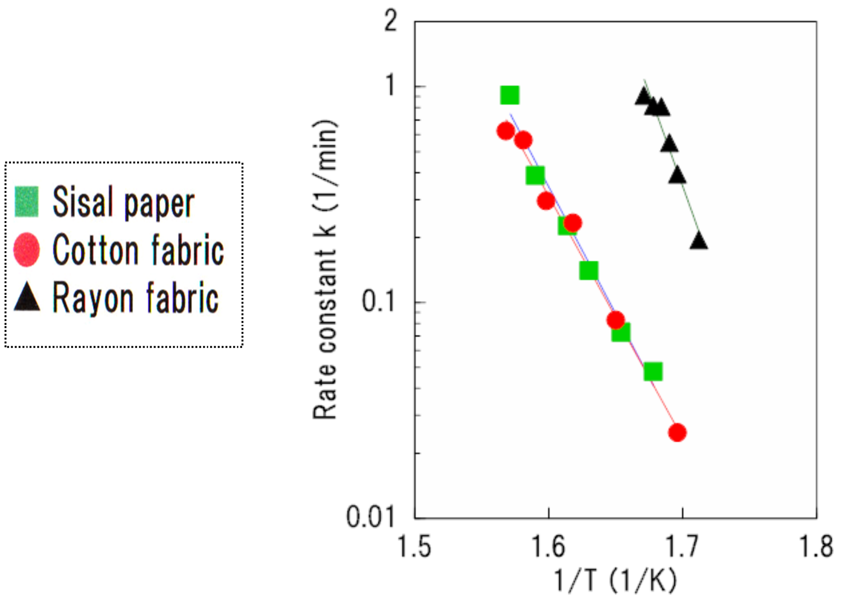

Figure S1 (I) Arrhenius plots for the carbonization reaction of sisal paper and cotton and rayon fabrics.

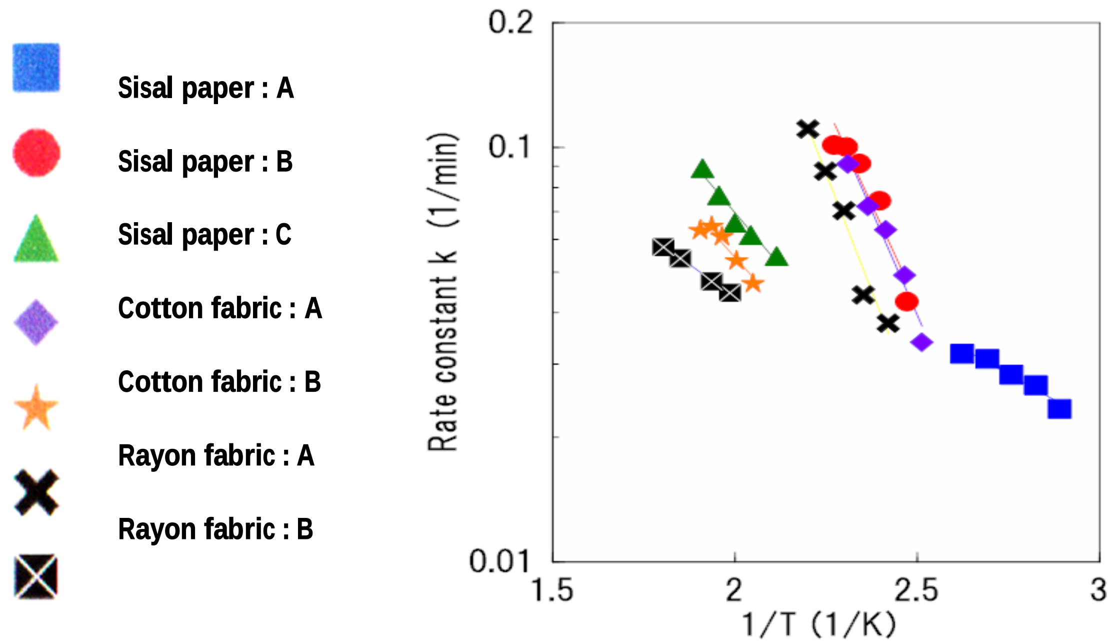

Figure S1 (Ⅱ) Arrhenius plots for the carbonization reaction of MSA-treated sisal paper and cotton and rayon fabrics. Symbols "A, B, and C" correspond to the temperature range shown in Table S3.

Table S3. Amount of absorbed MSA, carbon yield, and apparent activation energy.

	Absorbed MSA	Carbon yield	Temperature range	Apparent activation energy
	wt %	wt %	℃	KJ/mol
Sisal paper	0	11	320 - 365	225
Sisal paper treated with MSA	61	41	A 73 - 110	9
			B 130 - 165	34
			C 190 - 240	21
Cotton fabric	0	10.1	316 - 360	215
Cotton fabric treated with MSA	28	38	A 130 - 170	12
			B 220 - 260	16
Rayon fabric	0	10.6	310 - 326	330
Rayon fabric treated with MSA	26	37	A 145 - 184	42
			B 230 - 282	7

S5. Characterization of the two-dimensional (2D) carbon materials.

The thickness and basic weight of the 2D carbon materials prepared from the MSA-treated sheet-like cellulosic materials were measured using a thickness meter (Litematic VL-50A, Mitsutoyo) and a balance meter (AB-135-S, Mettler Toledo), respectively.

Elemental analysis of the 2D carbon materials was carried out using a Yanaco CHN Corder MT-5 device at the Microanalysis Center of the Kyoto University, as described in Section S2 of Supporting Information. The results are listed in Table S4.

The elemental analysis of the 2D carbon materials prepared at 800°C revealed the presence of sulfur of less than 1 wt% because MSA was used as a catalyst. However, the carbon materials heat-treated at temperatures above 2000°C exclusively contained elemental carbon.

Table S4. Characterization of the 2D carbon samples prepared at 800°C from the MSA-treated sheet-like cellulosic materials.

	Thickness (μm)	Basic weight (g/m^2)	Elemental analysis (wt %)		
			Carbon	Hydrogen	Nitrogen
Sisal paper	304	85	81.4	1.1	0.0
Cotton fabric	264	106	86.2	1.1	0.0
Rayon fabric	73	43	80.3	1.2	0.1

1. Thickness was calculated as the average value obtained by measuring 50 points of a specimen 100 mm ×× 100 mm in size.
2. Basic weight was calculated as the average value of 10 specimens 80 mm × × 100 mm in size and the measured thickness.

S6. ESR spectroscopy analysis of the 2D carbon materials.

Electron spin resonance (ESR) spectra of the 2D carbon materials were obtained on an ESR spectrometer from JEOL (Japan) (1 mW, @9.42 GHz).

The spectra of the 2D carbon samples prepared at 800°C from all the MSA-treated sheet-like cellulosic materials showed nearly the same line shapes, which were very sensitive to the heat-treatment temperature,[10] as exhibited in Figure S2. The 2D carbon materials heat-treated at 2600°C (Figure S2(c)) showed a typical Dyson type spectrum.[11] These results reveal that the 2D carbon materials have unpaired electrons. Thus, the carbon materials obtained in this work possess large amounts of hexagonal carbon-bond sheets in their free radical state.

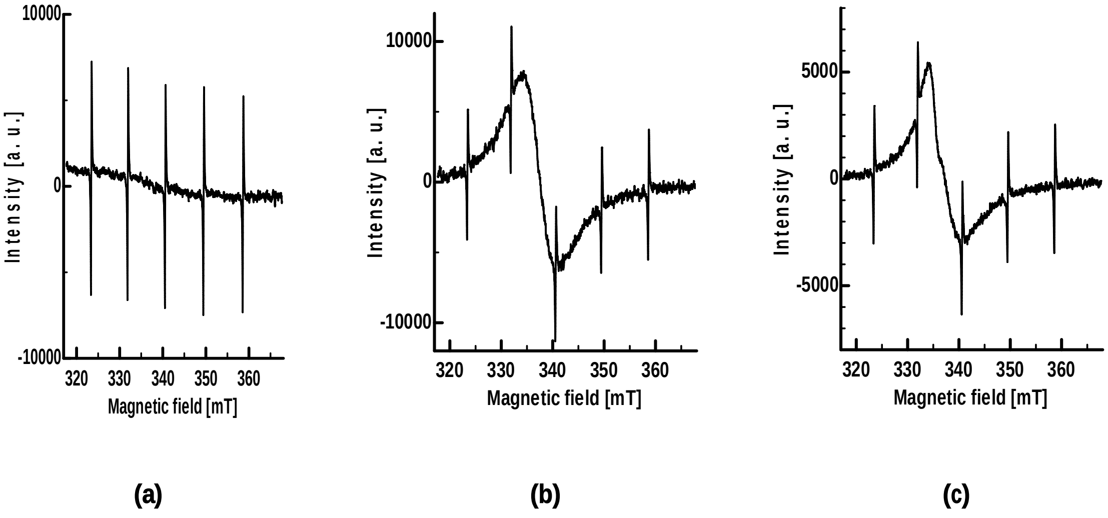

Figure S2. ESR spectra for the 2D carbon material prepared at 800°C from the MSA-treated cotton fabric (a) and those heat-treated at 1800°C (b), and at 2600°C (c) after the carbonization at 800°C. Mn^{2+}/MgO (a marker) was used as a standard for the calculation of g-value.

S7. Raman spectroscopy analysis of the 2D carbon materials

The Raman scattering spectra of the 2D carbon materials were obtained at room temperature by the back scattering method with a triple monochromator and a charge-coupled device detector on a Raman scattering spectroscope (NRS-2100, Jasco). A 100 mW argon ion laser with a wavelength of 514.5 mm was used.

Figure S3 shows the Raman scattering spectra of the 2D carbon materials prepared at 800°C (black curves) and those heat-treated at 2600°C (red curves).

The spectrum at the bottom (black curves) of Figure S3 showed a strong and broad peak at 1350 cm^{-1} attributed to the disordered structure (D-band) together with a comparable peak corresponding to the sp^2 hexagonal carbon-bond network (G-band) at 1580 cm^{-1} for all 2D carbon materials.[12–14] These two peaks became sharper with the heat-treatment temperature. At 2200 and 2600°C, the peak of the G-band was more intense than that of the D-band. These results suggest that very small fragments of carbon with sp^2 hexagonal network bonds were formed in the carbon materials prepared at 800°C, and these small fragments became larger and more ordered with the heat-treatment temperature, as described in a previous paper.[15]

It should be noted that a strong peak was observed at ca. 2700 cm^{-1} for all the heat-treated materials (red curves in Figure S3). It is believed that this peak corresponds to the second-order of the D-band and is very sensitive to the graphite structure along the c-axis.[13] Therefore, the peak at ca. 2700 cm^{-1} indicated that the 2D carbon materials heat-treated at 2600°C have large amounts of fragments with a graphitic structure.

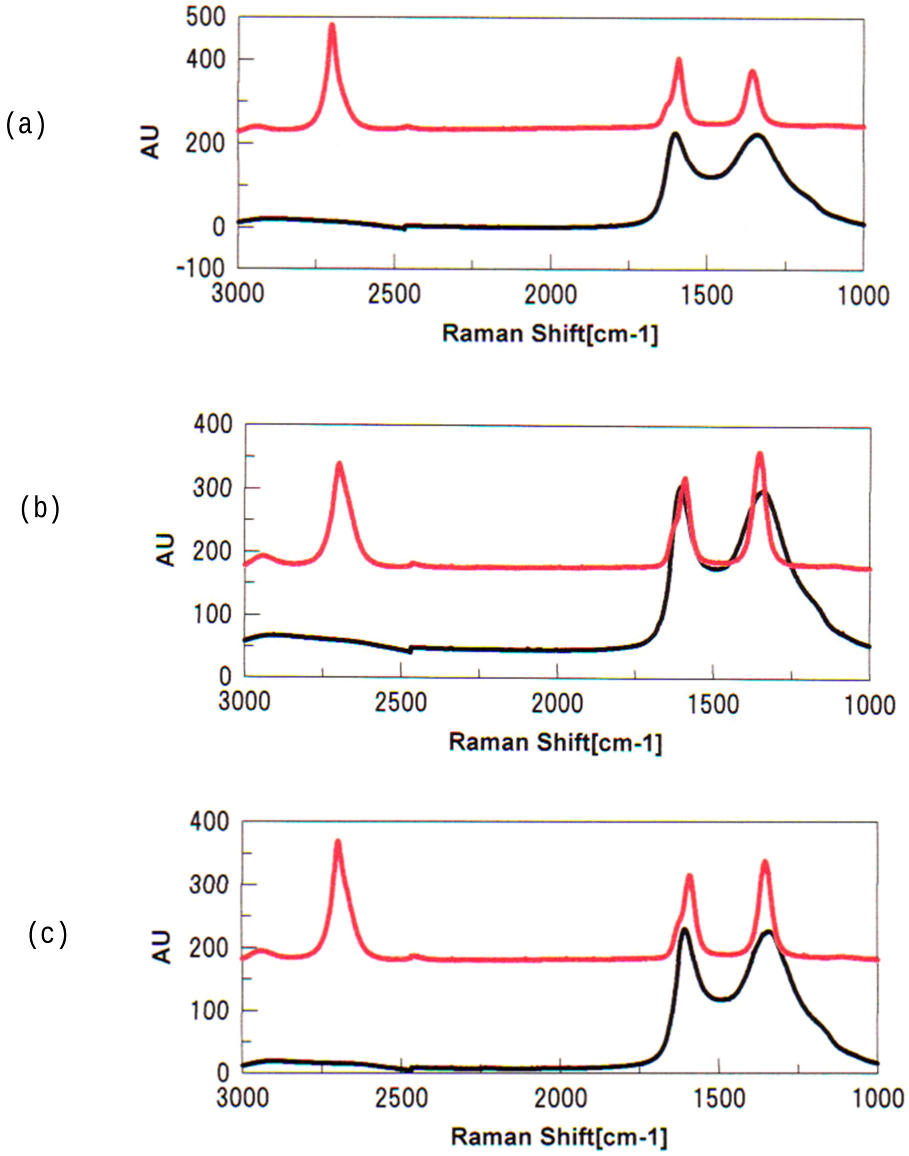

Figure S3. Raman scattering spectra of the 2D carbon materials prepared from MSA-treated sisal paper (a), cotton fabric (b), and rayon fabric (c). The black curves indicate the materials carbonized at 800°C, while the red curves correspond to the materials heat-treated at 2600°C after carbonization at 800°C.

S8. Mechanical properties of the 2D carbon materials

After carbonization through pyrolysis, the sheet-like cellulosic materials used in this work showed low carbon yields. The carbonized materials prepared were fragile, and therefore, their mechanical properties were difficult to measure. Conversely, the carbon materials prepared from the MSA-treated cellulosic materials showed high carbon yields and enhanced mechanical properties. Therefore, they can be regarded as novel 2D carbon materials from a macroscopic point of view.

The bending modulus E and the strength S of the 2D carbon materials were examined on a Tensilon RTG-1210 device (A & D). A tape-shaped specimen 15 mm in width and 60 mm in length was prepared for examination of its E and S values. The prepared specimen was placed in the Tensilon device. Both sides of the tape-shaped specimen were fixed to obtain a stress–strain curve. The E and S values were calculated using the following formula:[16]

$$E = (W L^3) / (16bt^3 \varepsilon)$$
$$S = (3 W L) / (2bt^2)$$

where, W denotes the stress applied to the specimen at break down and L is the length of the specimen. In this work, L was 40 mm. The specimen width was denoted by b and the thickness by t. . ε corresponds to the elongation of the specimen at break down. The calculation of E assumed that the cross section bending moment I of the specimen was given by $I = (b\ t^3)/12$. The W and ε values were obtained from the stress–strain curve of each specimen.

The E and S values obtained are listed in Table S5. Each value including thickness was obtained as the average of five specimens. The E and S values of the carbon materials prepared at 800℃ from the sisal paper (300 in thickness) were 3 GPa and 31 MPa, respectively. However, in the case of the carbon materials prepared from the sisal paper used in this work, the variation of the thickness was too large to reliably determine the mechanical properties of the heat-treated carbon materials.

Both E and S increased with the heat-treatment temperature for the 2D carbon materials prepared from the cotton (Table S5 (1)) and rayon fabrics (Table S5 (2)). This result can be explained by the improved crystallinity of the carbon materials with the heat-treatment temperature, as shown in Figure 3-2 in the main text. It should be noted that the 2D carbon materials prepared from the rayon fabrics (Table S5 (2)) showed remarkably high E and S values as compared with cotton fabrics. This difference in E and S between the two kinds of 2D carbon materials is believed to originate from the different compositions of the fabrics (spun yarns versus filaments). A carbon fiber prepared from a MSA-treated rayon filament is expected to present higher E and S values than that prepared from a spun yarn since the former fiber has a long continuous carbon microstructure different from that of the spun yarn.

Table S5

(1) Bending modulus and strength of the 2D carbon materials prepared from a cotton fabric

Heat-treatment temperature	Thickness	Strength	Modulus
	μm	MPa	GPa
800	257	48	5
1100	255	85	8
1400	257	82	8
1800	241	86	9
2200	248	84	9
2600	227	93	10

(2) Bending modulus and strength of the 2D carbon materials prepared from a rayon fabric

Heat-treatment temperature	Thickness	Strength	Modulus
℃	μm	MPa	GPa
800	81	157	75
1100	77	360	134
1400	70	577	326
1800	68	594	410
2200	67	697	454
2600	64	770	592

S9. Electrical properties of the 2D carbon materials

The electrical conductivity of the carbon materials depends on the degree of crystallinity and the crystal size. It is well known that carbon materials with a highly crystalline structure have high electrical conductivities.[17, 18]

The electrical conductivities of the 2D carbon materials were investigated on a low resistivity meter Loresta GP (Dia Instruments). Figure S4 shows the electrical conductivities of the 2D carbon materials used in this work as a function of the heat-treatment temperature. The conductivity of the 2D carbon materials prepared at 800℃ was 2–5 S/cm, and it significantly increased with the temperature of the heat treatment up to 1800℃ (20–26 S/cm) and levelled off thereafter, as shown in Figure S4. The 2D carbon materials prepared at 800℃ were mostly amorphous (see X-ray diffraction patterns in Figure 3-2), thereby explaining their low electrical conductivity. Likewise, the 2D carbon materials prepared at 2600℃ showed higher conductivity values because of their higher degree of crystallinity.

The electrical properties of the carbon materials prepared from other types of celluloses were investigated. Conducting carbon clusters were prepared from microcrystalline cellulose, as reported by Rhim et al.[17] They reported that the formation and growth of the carbon clusters enhanced with the heat-treatment temperature, thereby increasing the electrical conductivity. In fact, the conductivity increased with the heat-treatment temperature and reached a plateau at temperatures higher than 1200℃. Such a change in the conductivity was consistent with the behavior of the 2D carbon materials observed in this study.

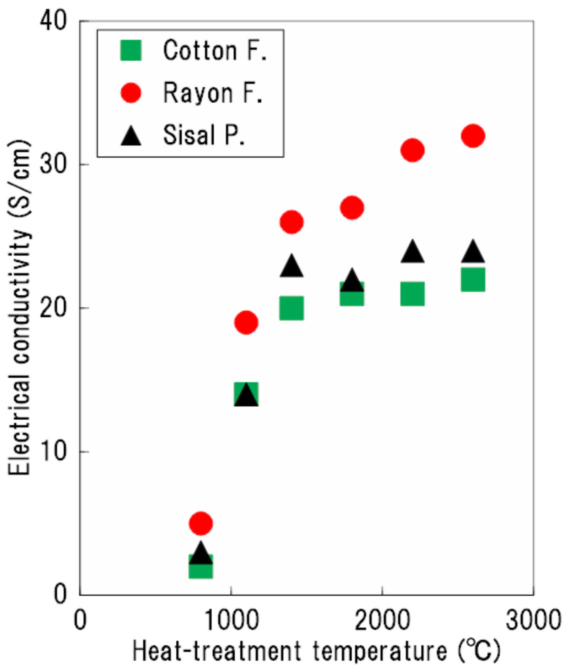

Figure S4. Electrical conductivity of the 2D carbon materials prepared at 800℃ from the sisal paper (▲), cotton fabric (■), and rayon fabric (●)as a function of the heat-treatment temperature.

S10. Pore size and micro-void distributions for the 2D carbon materials.

The 2D carbon materials obtained in this work were porous and had a large number of gas permeable micro-voids.[19] The porous structure is essential for the application of 2D carbon materials in electrodes, serving as a gas diffusion layer (GDL) in a polymer electrolyte fuel cell (PEFC).[20] The pore size distribution of the 2D carbon materials as a GDL has an effect on the output power of the PEFC.

The pore size and micro-void distributions of the 2D carbon materials used in this work were investigated using a pore size distribution meter Porometer 3G (Quantachrome Instruments). The pore size distribution curves of the 2D carbon materials are shown in Figure S5. In the case of the 2D carbon material prepared from the sisal paper, a normal distribution curve with a maximum at 23 μm in pore size was obtained (Figure S5 (a)). This result reveals that the axes of the fibrous structures were randomly situated and had no preferred orientation in the 2D carbon materials. On the other hand, the curves were asymmetrical for the carbon materials prepared from both cotton and rayon fabrics, as shown in Figures S5 (b) and S5 (c), respectively. These results likely indicate that the fabrics were composed of both warp and weft yarns with different thicknesses. The 2D carbon material prepared from the rayon fabric had yarns composed of very thin multi-carbon fibers. This is a reason why this sample showed a wider pore size distribution as compared to the rest of 2D carbon materials.

(a)
(b)
(c)

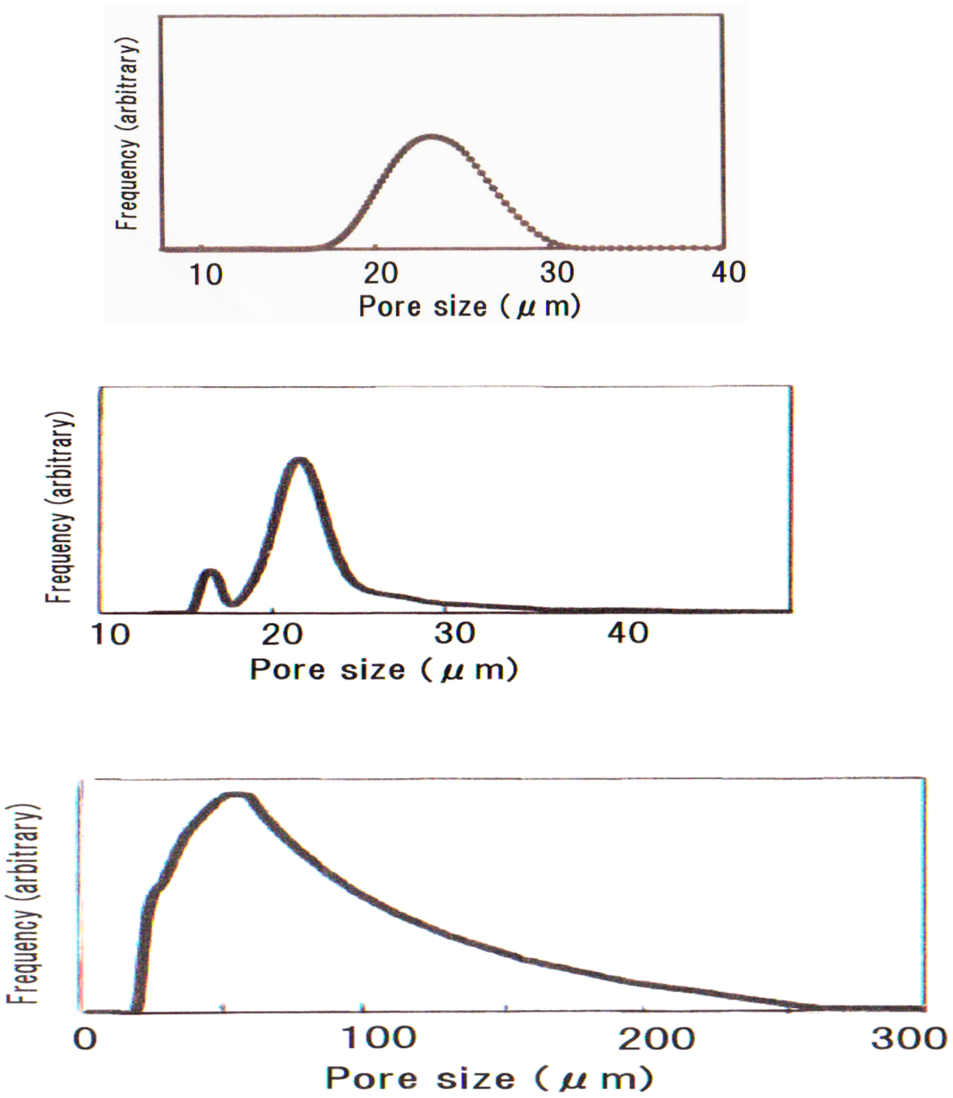

Figure S5. Pore size and micro-void distributions for the 2D carbon materials prepared at 800°C from the sisal paper (a), cotton fabric (b), and rayon fabric (c).

S11. Example of application of the 2D carbon materials: a GDL for polymer electrolyte fuel cells.

Taking advantage of the high electrical conductivity (20–33 Scm^{-1} in the surface lateral direction), porous morphology, softness, and smooth surface structure characteristics of the 2D carbon materials obtained in this work. The 2D carbon materials are promising components of GDL to be used in low temperature fuel cells.[21, 22] When compared with commercial GDLs usually made from rigid and straight poly(acrylonitrile)-derived carbon fiber sheets, the 2D carbon materials cost lower and are easier to fabricate while also being compatible with nano-Pt particles supported on carbon powder (Pt/C) catalyst layers.

Although the 2D carbon samples prepared from cellulosic materials showed lower electrical conductivity as compared with commercial carbon fiber sheets, the former presented better pore structure and surface morphology, as shown in Photo S1. The 2D carbon materials can serve as a support for the catalyst layer while also having good adhesion with proton conducting polymer films (e.g., Nafion® membranes). When comparing the performances of this GDL with those of commercial GDL materials, the former materials showed equal or even better power generation performances (Table S6). These results show that this eco-material could be a promising candidate for being used in power generation devices, playing a central role in the future hydrogen energy society by being applied in fuel cell vehicles and on-site power generation devices.

A membrane electrode assembly (MEA) was prepared with a 20 wt% Pt/C catalyst (ElectroChem, EC-20-PTC), Nafion 115 membrane (DuPont N-115, 125μm), and the 2D carbon material as a GDL, and subsequently tested for fuel cell operation. The MEA was a pile composed of anode GDL/H2 oxidation catalyst layer/proton conducting polymer film/O_2 reduction catalyst layer/cathode GDL, and was hot-pressed at 135°C, 100 kg cm^{-2} for 3 min. As a reference, a commercial GDL 0.3 mm in thickness was also used. The Pt catalyst loading in a 4 cm2 electrode area MEA was 0.6×10^{-3} g/cm2 both for the anode and the cathode. The MEAs were incorporated in a single fuel cell test equipped with serpentine H_2 and O_2 gas flow fields, and tested in the H_2/O_2 mode at 70°C, with an automated measuring assembly (potential scan rate of 0.12 V/min). H_2 and O_2 gasses were humidified at 60°C, with a flow rate of 50 and 100 ml/min, respectively.

Potential–current and power density–current relations revealed good performance in the fuel cell power generation modes comparable with or better than those containing a commercial GDL as shown in Figures S6 (a)–(c).

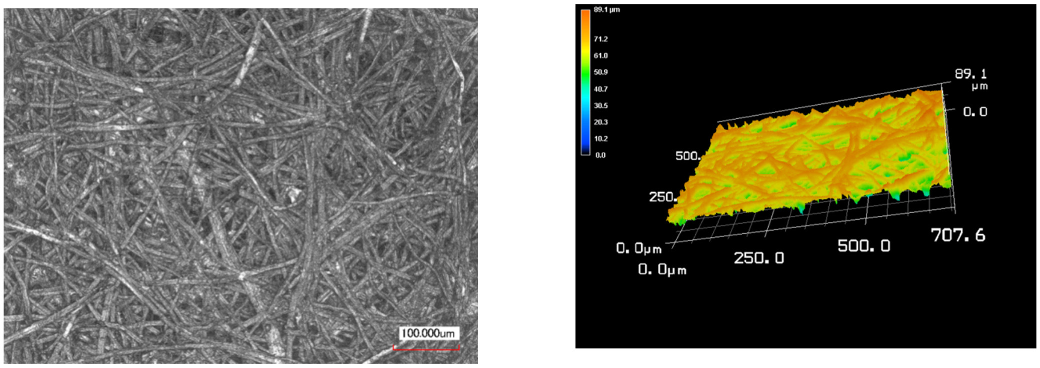

(a)

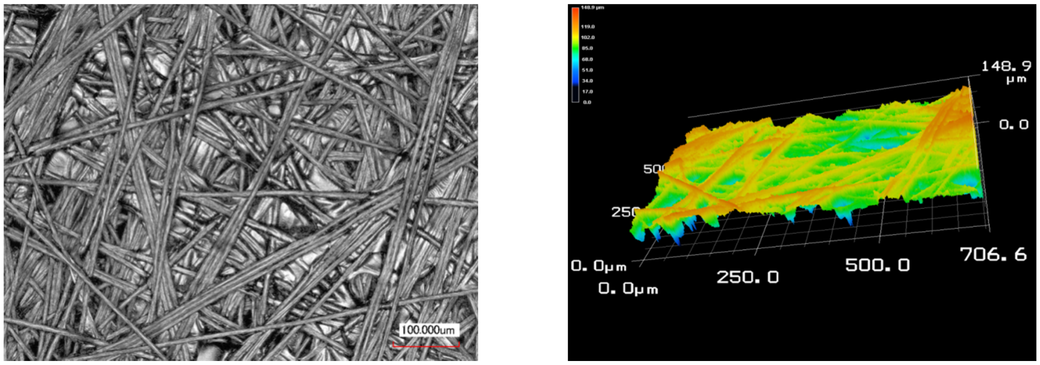

(b)

Photo S1. SEM image (left side) and laser microscopic view (right side) of a GDL made from sisal paper (a) and a commercial GDL (b). A VK9700 microscope (KEY- ENCE Co.) was used as a laser-microscope (courtesy of KEYENCE).

(a)

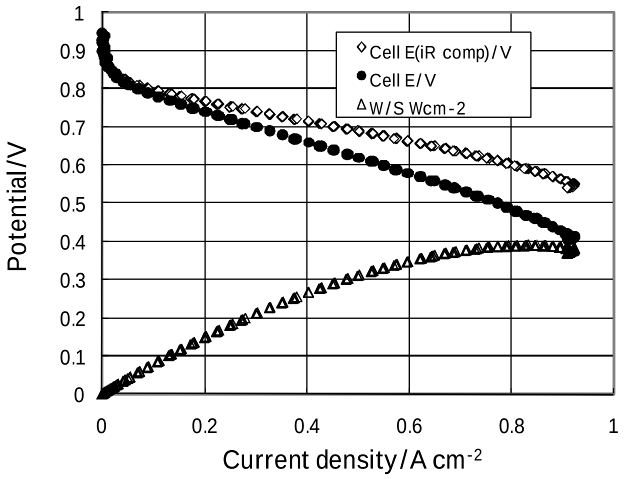

(b)

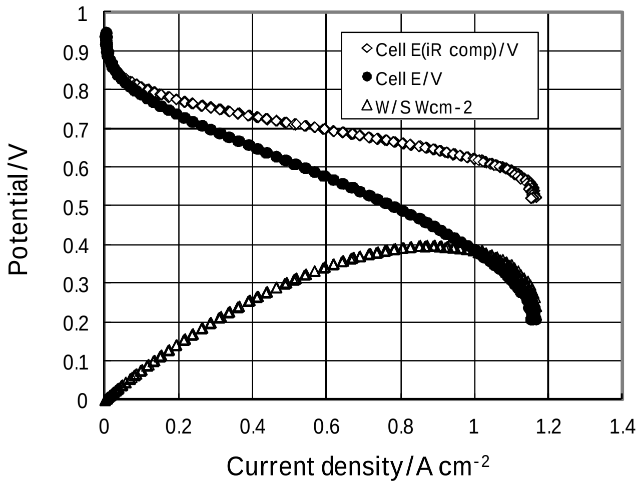

(c)

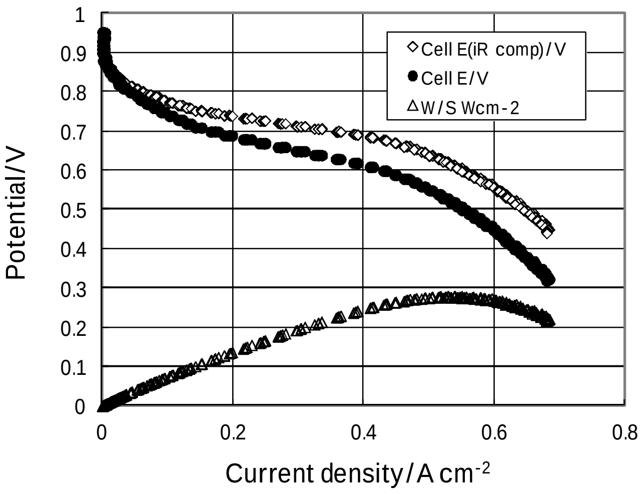

Figure S6 Potential and power density vs. current density curves of the MEA using a 2600°C heat-treated 2D carbon material prepared from sisal paper (a) and a 1800°C heat-treated 2D carbon material prepared from cotton fabric (b) as GDLs, and a commercial GDL (Toray TGP-H-090) (c). ●: uncompensated potential, ○: ohmic resistance compensated potential, △: cell power.

Table S6. Peak power densities of the fuel cells containing the MEAs using heat-treated 2D carbon materials prepared from sisal paper and cotton fabric as GDLs together with a commercial GDL (Toray TGP-H-090).

2D carbon material	Heat-treatment temperature (℃)	Peak power density (W / cm^2)
Prepared from sisal paper	800	0.15
	1400	0.27
	1800	0.33
	2200	0.34
	2600	0.39
Prepared from cotton fabric	800	0.15
	1100	0.39
	1800	0.40
Commercial GDL		0.28

References to SI.

[1] A. R. Martin, M. A.Martin, O. R. R. F. da Silva, L. H. C. Mattoso, *Thermochim. Acta.* **2010**, *506*, 14.

[2] J. I. Morán, V. A. Alvarez, V. P. Cyras, A. Vázquez, *Cellulose.*-**2008,** *15*, 149.

[3] Canadian Standard Freeness (CSF), *TAPPI Test Method T 227 om-09*. **2009**

[4] V. Mamleev, S. Bourbigot, M. Le Bras, J. Yvon, *J. Anal. Appl. Pyrol.*-**2009**, *84*, 1.

[5] M. V. Ramiah, *J. Appl. Polym. Sci.*-**1970**, *14*, 1323.

[6] J. E. White, W. J. Catallo, B. L. Legendre, *J. Anal. Appl. Pyrol.*-**2011**, *91*, 1.

[7] F. Shafizadeh, *J. Polym. Sci. Polym. Symposia.*-**1971**, *36*, 21.

[8] M. J. Antal, G. Varhegyi, *Ind. Eng. Chem. Res.* **1995**, *34*, 703.

[9] V. Mamleev, S. Bourbigot, M. L. Bras, J. Yvon, J. Lefebvre, *Chem. Eng. Sci.* **2006**, *61*, 1276.

[10] J. B. Jones, L. S. Singer, *Carbon.* **1982,** *20*, 379.

[11] G. Wagoner *Phys. Rev.* **1960**, *118*, 647.

[12] M. Inagaki, *New carbons-control of structure and functions.* Elsevier, UK **2000**.

[13] M. A. Pimenta, G. Dresselhaus, M. S. Dresselhaus, L. G. Cancado, A. Jorio, R. Saito, *Phys. Chem. Chem. Phys.* **2007,** *9*, 1276..

[14] A. C. Ferrari, D. M. Basko, *Nature Nanotech.* **2013,** *8*, 235.

[15] M. Kyotani, S. Matsushita, S. Kimura, K. Akagi, *J. Anal. Appl. Pyrol.* **2012**, *95*, 14.

[16] S. Timoshenko, *Elements of Strength of Materials.* Van Nostrand, USA, **1968**.

[17] Y. Rhim, D. Zhang, D. H. Fairbrother, K. A. Wepasnick, K. J. Livi, R. J. Bodnar, D. C. Nagle, *Carbon.* **2010**, *48*, 1012.

[18] L. J. Kennedy, J. J. Vijaya, G. Sekaran, *Mater. Chem. Phys.* **2005**, *91*, 471.

[19] Y. Liang, B. Wu, D. Wu, F. Xu, Z. Li, J. Luo, H. Zhong, R. Fu, K. Matyjaszewski, *J. Mater. Chem.* **2011**, *21*, 14424.

[20] E. Gauthier, Q. Duan, T. Hellstern, J. Benziger, *Fuel Cells.* **2012,** *12*, 835.

[21] M. F. Mathias, J. Roth, J. Fleming, W. Lehnert, Diffusion media materials and characterization, Chap. 42, Vol. 3, in Handbook of Fuel Cells. (Lamm, A. & Gasteiger H. A. Eds.) Wiley, **2003.**

[22] O. M. Orogbemi, D. B. Ingham, M. S. Ismail, K. J.Hughes, L. Ma, *Hydrogen Energy*. **2016**, *41*, 21345..
